# Supplementary material for: A Pilot Study to Investigate the Balance between Proteases and α1-Antitrypsin in Bronchoalveolar Lavage Fluid of Lung Transplant Recipients
Source: High Throughput. 2019 Feb 13;8(1):5. doi: 10.3390/ht8010005 (PMC6480715; doi:10.3390/ht8010005)
Supplement: Supplementary file 1 [file high-throughput-08-00005-s001.pdf]

ACCESSION	MASS	SCORE(%)	COVERAGE(%)	DESCRIPTION	M/Z	Z	PEPTIDE
sp|P01009|	46,737	99	14,83%	Alpha-1-antitrypsin OS=Homo sapiens			
	1345,9855				674	2	RLGMFNIQHCK
	920,99274				922	1	FLENEDR
	1777,9855				890	2	TDTSHHDQDHPTFNK
	1331,9783				445	3	LVDKFLEDVKK
	1077,9855				540	2	FLENEDRR
	1544,9783				516	3	SPLFMGKVVNPTQK
	851,9855				427	2	SASLHLPK
	1317,9855				660	2	LGMFNIQHCKK
sp|P08246|	28,518	62	4,87%	Neutrophil elastase OS=Homo sapiens			
	1545,9855				774	2	AVRVVLGAHNLSRR
	1389,9855				696	2	AVRVVLGAHNLSR

Table S1. Primary sequence of all peptides identified for each protein

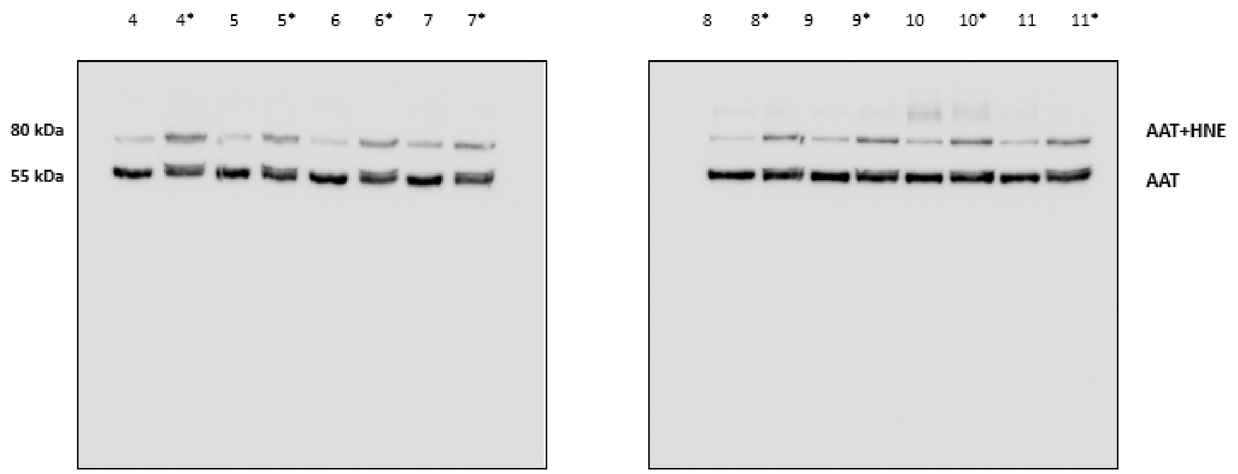

Figure S1- Western blotting of BALf from patients with the 80 kDa band before and after incubation with exogenous HNE* (left to right in both panels).
